# Avaliação de Risco em Insuficiência Cardíaca: Abrangente é Sempre Melhor!

**DOI:** 10.36660/abc.20230760

**Published:** 2023-12-14

**Authors:** Miguel Mendes

**Affiliations:** 1 Centro Hospitalar de Lisboa Ocidental EPE Hospital de Santa Cruz Lisboa Portugal Centro Hospitalar de Lisboa Ocidental EPE – Hospital de Santa Cruz – Cardiologia, Lisboa – Portugal

**Keywords:** Insuficiência Cardíaca Sistólica, Medição de Risco/métodos, Teste de Esforço Cardiopulmonar/métodos, Classificação/New York Heart Association, Clasiificação de Weber

A avaliação de risco na insuficiência cardíaca (IC) é muito desafiadora, abrangendo muitos dados, como classe NYHA, história clínica, comorbidades, parâmetros de testes clínicos, marcadores bioquímicos, adesão e tolerância aos medicamentos recomendados pelas diretrizes. ^1 , 2^

A avaliação do risco é fundamental na IC avançada para apoiar a decisão de fornecer a terapia mais adequada para um determinado paciente, desde o transplante cardíaco até o DAVi de longa duração ou cuidados paliativos. ^1 - 3^

Vários sistemas de escore, como o Heart Failure Survival Score (HFSS), Seattle Heart Failure Score (SHFM), Metabolic Exercise Cardiac Kidney Index (MECKI) e Meta-analysis Global Group Chronic Heart Failure (MAGGIC), demonstraram ser insatisfatórios, particularmente no grupo de pacientes de alto risco. Os parâmetros do teste de exercício cardiopulmonar (TECP) são considerados no HFSS (pico VO _2_ ) e no escore MECKI (pico previsto VO _2_ e VE/VCO _2_ slope; a classe NHYA integra SHFM e MAGGIC. ^4 - 6^

Pedro Engster et al. em “ Papel Incremental da Classificação da New York Heart Association e dos Índices do Teste de Exercício Cardiopulmonar para Prognóstico na Insuficiência Cardíaca: um Estudo de Coorte”, ^7^ publicado nesta edição, avaliou o valor agregado para avaliação de risco da classificação subjetiva NHYA à classificação objetiva de Weber, com base no valor do VO _2_ pico. Estudaram uma população adulta com IC (n=834), avaliada em um centro terciário brasileiro, com fração de ejeção (FE) inferior a 50% (FE mediana = 32%), 30% com etiologia isquêmica, sob os medicamentos para IC recomendados nas diretrizes, bem equilibradas entre ambos os sexos (42% mulheres) e classes NHYA, exceto classe IV da NYHA (apenas 29 pacientes).

Encontraram um ganho na avaliação prognóstica para o risco de mortalidade por todas as causas quando os dois tipos de dados são considerados em conjunto.

A classe NYHA atribuída pelo médico e a classe Weber derivada do TECP foram estratificadas em “favorável” (NYHA I ou II e Weber A ou B) ou “adversa” (NYHA III ou IV e Weber C ou D). Pacientes com uma classe favorável e uma classe adversa foram definidos como “discordantes”.

Eles também estudaram o impacto das classificações favoráveis e adversas para o VE/VCO _2_ *slope* e o percentual previsto do VO _2_ pico (PPVO _2_ ), classificando os pacientes como favoráveis quando o VE/VCO _2_ slope era inferior ou igual a 36 e o PPVO _2_ era igual ou superior a 50%, e como adversos quando VE/VCO _2_ e PPVO _2_ , foram respectivamente superiores a 36 ou inferiores a 50%.

Como esperado, descobriram que os pacientes com perfil favorável (classes I-II do NHYA e classes A e B de Weber) tinham melhores prognósticos do que os pacientes com perfil adverso (classes NYHA III-IV e classes C e D de Weber). Em uma análise multivariada, um aumento de uma classe da NYHA e uma diminuição de 3ml/Kg/min no pico VO _2_ aumentaram significativamente a mortalidade em 50%.

Nos 299 pacientes com classificação discordante foi encontrado prognóstico intermediário. O alargamento da análise aos valores do PPVO _2_ e da VE/VCO _2_ *slope* não alterou significativamente a avaliação prognóstica, ao contrário do que foi encontrado em muitos artigos publicados, nomeadamente no que diz respeito à VE/VCO _2_ *slope* , a quem tem atribuído um elevado impacto prognóstico.

Os autores concluíram que a classe NYHA e as medidas do TECP atribuídas pelo médico fornecem informações prognósticas complementares, mostrando que ambos os parâmetros têm impacto prognóstico independente.

A classe NYHA, por ser subjetiva, é frequentemente criticada, mas mostrou-se neste manuscrito útil nos pacientes “discordantes”, onde um risco intermediário poderia ser definido.

As conclusões deste manuscrito devem ser consideradas com cautela. A classe NYHA atribuída é resultado da estimativa subjetiva das limitações clínicas percebidas pelos pacientes e pelo médico. ^8^ Está sujeito à variabilidade interindividual (paciente) e interobservador (médico). Depende do psiquismo do paciente e do nível de atividade física habitual, que pode diminuir ou aumentar as queixas, e da percepção do médico sobre o caso. Por outro lado, os médicos, muitas vezes, têm dificuldade em escolher uma classe da NYHA para um determinado paciente. É comum encontrar classificações como I-II, II-III e III-IV em prontuários. A classificação das classes II e III da NYHA aos pacientes deste trabalho pode ter sofrido dificuldades e imposto erros de classificação.

Em relação à classificação de Weber, ^9^ algum erro de classificação dos pacientes também pode ter ocorrido, uma vez que os autores não demonstraram que apenas os pacientes que atingiram um VO _2_ máximo, confirmado pela obtenção de um platô ou queda de VO _2_ no pico do exercício, ou um valor de pico da relação de troca respiratória acima de 1,10, um substituto do VO _2_ máximo ou quase máximo foi incluído. Além disso, a classificação de Weber não leva em consideração o valor do PPVO _2_ em função da idade, sexo e massa corporal magra, classificando, consequentemente, na mesma classe pacientes com diferentes graus de aptidão cardiorrespiratória (ACR). ^10^ Na verdade, a ACR é melhor definida pelo pico VO _2_ , que é uma variável contínua (não categórica) reconhecida para estratificação de risco juntamente com outros parâmetros do TECP ^11^ e na IC avançada, particularmente quando um valor de pico VO _2_ abaixo de 12 ou 14 mL/Kg/min foi alcançado, respectivamente para pacientes em uso ou não de betabloqueadores. ^1 , 2^

Em conclusão, Engster et al., ^7^ demonstraram que considerar em conjunto os dados das classificações NYHA e Weber pode ser um primeiro passo para estratificação de risco em insuficiência cardíaca reduzida ou levemente reduzida. Esta abordagem restritiva deve ser enriquecida pela inclusão de outros parâmetros e biomarcadores para ser mais preciso e clinicamente útil.
