# Supplementary material for: Avaliação de Risco em Insuficiência Cardíaca: Abrangente é Sempre Melhor!
Source: Arq Bras Cardiol. 2023 Dec 14;120(11):e20230760. [Article in Portuguese] doi: 10.36660/abc.20230760 (PMC10789193; doi:10.36660/abc.20230760)
Supplement: Translation

# Translation: Risk Assessment in Heart Failure: Comprehensive is Always Better!

**Authors:** Miguel Mendes

**Affiliations:** 1 Portugal Centro Hospitalar de Lisboa Ocidental EPE – Hospital de Santa Cruz – Cardiologia, Lisboa – Portugal

**Keywords:** Heart Failure, Systolic, Risk Assessment/mehods, Cardiopulmonary Exercise Test/methods, Classification/NYHA, Classification Weber

Risk assessment in heart failure (HF) is very challenging, encompassing many data, like NYHA class, clinical history, comorbidities, clinical test parameters, biochemical markers, adherence, and tolerance to guideline-recommended drugs. ^1 , 2^

Risk assessment is critical in advanced HF to support the decision to provide the most adequate therapy for a given patient, from heart transplantation to long-duration LVAD or palliative care. ^1 - 3^

Several scoring systems, like the Heart Failure Survival Score (HFSS), Seattle Heart Failure Score (SHFM), Metabolic Exercise Cardiac Kidney Index (MECKI), and Meta-analysis Global Group Chronic Heart Failure (MAGGIC), demonstrated to be unsatisfactory, particularly in the high-risk group of patients. Cardiopulmonary exercise test (CPET) parameters are considered in HFSS (peak VO _2_ ) and MECKI score (predicted peak VO _2_ and VE/VCO _2_ slope); the NHYA class integrates both SHFM and MAGGIC. ^4 - 6^

Pedro Engster et al. in “Incremental Role of New York Heart Association Class and Cardiopulmonary Exercise Test Indices for Prognostication in Heart Failure: A Cohort Study”, ^7^ published in this issue, studied the added value for risk assessment of the subjective NHYA classification to the objective Weber classification, based on the value of peak VO _2_ . They studied an adult HF population (n=834), assessed in a tertiary Brazilian center, with an ejection fraction (EF) below 50% (median EF = 32%), 30% with ischemic etiology, under the HF drugs recommended in the guidelines, well-balanced between both genders (42% female) and NHYA classes, except for NYHA class IV (only 29 patients).

They found a gain in prognostic assessment for all-cause mortality risk when both types of data are considered together.

The physician-assigned NYHA class and the CPET-derived Weber class were stratified into “favorable” (NYHA I or II and Weber A or B) or “adverse” (NYHA III or IV and Weber C or D). Patients with one favorable class and one adverse class were defined as “discordant.”

They also studied the impact of favorable and adverse classifications for VE/VCO _2_ slope, and percent predicted peak VO _2_ (PPVO _2_ ), classifying the patients as favorable when VE/VCO _2_ slope was inferior or equal to 36, and PPVO _2_ was equal or superior 50%, and as adverse when VE/VCO _2_ and PPVO _2_ , were respectively superior to 36 or inferior to 50%.

As expected, they found that patients with a favorable profile (NHYA class I-II and Weber class A and B) had better prognoses than patients with an adverse profile (NYHA III-IV and Weber class C and D). In a multivariate analysis, an increase by one NYHA class and a decrease by 3ml/Kg/min in peak VO _2_ significantly increased mortality by 50%.

In the 299 patients with discordant classification, an intermediate prognosis was found. Enlarging the analysis to the values of PPVO _2_ and VE/VCO _2_ slope did not change the prognosis assessment significantly, contrary to what was found in many published papers, particularly regarding VE/VCO2 slope, to whom it has attributed a high prognostic impact.

The authors concluded that physician-assigned NYHA class and CPET measures provide complementary prognostic information, showing that both parameters have independent prognostic impact.

NYHA class, being subjective, is frequently criticized, but it showed in this manuscript to be useful in the “discordant” patients, where an intermediate risk could be defined.

This manuscript’s conclusions must be considered with caution. The attributed NYHA class is the result of subjective estimation of the clinical limitations perceived by the patients and by the doctor. ^8^ It is subject to inter-individual (patient) and inter-observer (physician) variability. It depends on the patient’s psychism and level of usual physical activity, which may decrease or increase the complaints, and the perception of the doctor to the case. On the other side, the clinicians several times, have difficulty choosing one NYHA class for a given patient. It is common to find classifications like I-II, II-III, and III-IV in medical records. The classification of II and III NYHA classes to patients in this paper may have suffered difficulties and imposed misclassification.

Concerning Weber classification, ^9^ some patient misclassification may also have happened since the authors did not demonstrate that only patients reaching a VO _2_ maximum, confirmed by the attainment of a VO _2_ plateau or drop at peak exercise, or a peak value of respiratory exchange ratio over 1.10, a surrogate of VO _2_ maximum or near-maximum were included. Besides this, Weber’s classification does not take into consideration the value of PPVO _2_ in function of age, gender, and lean body mass, classifying, consequently, in the same class patients with different degrees of cardiorespiratory fitness (CRF). ^10^ Indeed, CRF is best defined by peak VO _2,_ which is a continuous (not a categorical) variable recognized for risk stratification together with other CPET parameters ^11^ and in advanced HF, particularly when a value of peak VO _2_ below 12 or 14 mL/Kg/min was reached, respectively for patients on or without beta-blockers. ^1 , 2^

In conclusion, Engster et al. ^7^ demonstrated that considering together data from NYHA and Weber classifications may be a first step for risk stratification in reduced or mildly reduced heart failure. This restrictive approach must be enriched by including other parameters and biomarkers to be more accurate and clinically useful.
